# WormScan: A Technique for High-Throughput Phenotypic Analysis of *Caenorhabditis elegans*

**DOI:** 10.1371/journal.pone.0033483

**Published:** 2012-03-23

**Authors:** Mark D. Mathew, Neal D. Mathew, Paul R. Ebert

**Affiliations:** School of Biological Sciences, University of Queensland, St. Lucia Campus, Brisbane, Queensland, Australia; Centre for Genomic Regulation, Spain

## Abstract

**Background:**

There are four main phenotypes that are assessed in whole organism studies of *Caenorhabditis elegans*; mortality, movement, fecundity and size. Procedures have been developed that focus on the digital analysis of some, but not all of these phenotypes and may be limited by expense and limited throughput. We have developed WormScan, an automated image acquisition system that allows quantitative analysis of each of these four phenotypes on standard NGM plates seeded with *E. coli*. This system is very easy to implement and has the capacity to be used in high-throughput analysis.

**Methodology/Principal Findings:**

Our system employs a readily available consumer grade flatbed scanner. The method uses light stimulus from the scanner rather than physical stimulus to induce movement. With two sequential scans it is possible to quantify the induced phototactic response. To demonstrate the utility of the method, we measured the phenotypic response of *C. elegans* to phosphine gas exposure. We found that stimulation of movement by the light of the scanner was equivalent to physical stimulation for the determination of mortality. WormScan also provided a quantitative assessment of health for the survivors. Habituation from light stimulation of continuous scans was similar to habituation caused by physical stimulus.

**Conclusions/Significance:**

There are existing systems for the automated phenotypic data collection of *C. elegans*. The specific advantages of our method over existing systems are high-throughput assessment of a greater range of phenotypic endpoints including determination of mortality and quantification of the mobility of survivors. Our system is also inexpensive and very easy to implement. Even though we have focused on demonstrating the usefulness of WormScan in toxicology, it can be used in a wide range of additional *C. elegans* studies including lifespan determination, development, pathology and behavior. Moreover, we have even adapted the method to study other species of similar dimensions.

## Introduction

*Caenorhabditis elegans* is an ideal genetic model organism that has been applied to a wide range of studies into toxicology [1], lifespan [2], development, neurobiology [3] and pathology [4]. These studies rely on four main whole organism phenotypes; movement [5], [6], [7], [8], [9], [10], mortality [1], [11], fecundity [12], [13], [14] and size [15], [16]. While the small size of *C. elegans* enhances its utility as an *in vivo* model organism it also complicates scoring of phenotypes. The WormScan technique overcomes the experimental bottleneck associated with scoring phenotypes of large numbers of individuals.

Automated or high-throughput procedures have been developed to analyze movement [9], [17], mortality [18], fecundity [19] and size [20]. Death in *C. elegans* is manually determined by an animal's inability to respond to a mechanical stimulus [21], often by touching with a ‘worm pick’. This assay is labor intensive and repetitive, making it a prime candidate for automation. Additionally, current high-throughput methods require expensive or specialized equipment [17], [22], [23], [24], [25], [26].

To improve data acquisition in whole organism studies we have developed WormScan as a low-cost, high-throughput screening method based on a flatbed scanner. This procedure produces images of sufficient quality for robust identification of nematodes and allows a large numbers of culture plates to be processed in parallel. Scanners have previously enabled automated counting of mammalian and bacterial cell colonies, as well as virus plaques [27], [28], [29], [30]. Scanning does not allow the high-frame rate image capture of camera-based methods. However, most *C. elegans* publications rely on very simple behavioral assays, primarily changes in rate of movement [31], which is quantifiable by the WormScan method. A factor that makes the scanner particularly useful for analysis of behavioral response is that high intensity light stimulates is an aversive stimulus that triggers phototaxis [32], [33]. With two sequential scans of a plate of worms it is possible to determine the number of nematodes that respond to the light stimulus of the initial scan and even quantify the degree to which they move.

## Methods

### Nematodes

*C. elegans* were maintained under standard conditions at 20°C on NGM agar containing *E. coli* (OP50). Age-synchronized nematode cultures were derived from eggs harvested from adult *C. elegans* by exposure to bleach. Eggs were then left to hatch over-night in M9 buffer with aeration. Growth was initiated by feeding [34]. All assays were conducted with either the wild-type, Bristol isolate of *C. elegans* (N2) or the phosphine-resistant mutant, *pre-33* [35], [36] that was generated in the N2 background.

### Exposure to chemicals and phenotypic analysis

For assay, 9 ml of NGM agar was added to 5.5 cm petri plates to a depth of approximately 0.33 cm. Synchronized L1 nematodes were added to plates that had previously been seeded with OP50 bacteria. Phosphine gas was generated and *C. elegans* were exposed across a linear concentration range, at 20°C as previously described [35], [36], [37]. After a recovery period of 48 hours, movement in response to light stimulus, mortality, and length were quantified for all individuals on each plate of nematodes. Fecundity was determined without phosphine exposure as previously described [35]. Progeny nematodes were allowed to grow to the young adult stage at which time the nematodes were easily distinguished from scanning artifacts. Lifespan was also determined without phosphine exposure by transferring a single L4 stage *C. elegans* to each well of 12 well tissue culture plates containing NGM that had been seeded with OP50 and 40 µM 5-fluoro-2′-deoxyuridine.

### Image capture

An Epson Perfection V700 Photo Scanner was used for transmission scanning of *C. elegans* on agar plates. Other than the lifespan and fecundity experiments, nematodes to be scanned were cultured at a density of 30–150 individuals per 5.5 cm plate. Images were captured in 16-bit grayscale at a resolution of 2400 dpi and a rate of 2 frames/180 s. Scanned images were attained using Epson Scan software version 3.810. The dimensions of an image produced by the scanner were measured, which confirmed that the dpi rating matched the physical size of the generated image.

### Image analysis

Image analysis relies on the FIJI implementation of ImageJ (http://rsbweb.nih.gov/ij/ version 1.46a) with the following additional plugins; image stabilization (http://www.cs.cmu.edu/~kangli/code/Image_Stabilizer.html version 18/06/2010) and hysteresis (http://imagejdocu.tudor.lu/doku.php?id=plugin:filter:edge_detection:start version 22/3/2011). The plugins included in the FIJI package that were used are Advanced Weka Segmentation [38] (version 17/11/2011) and AnalyzeSkeleton [39]. All data analysis was performed on a computer with 2.8 GHz quad-core Intel Core i7 processor and 16 GB of RAM. A detailed tutorial and scripts are provided in Tutorial S1.

The first step in any of the WormScan procedures is to identify worms within the raw data image and to align sequentially scanned images. To begin, the image segmentation software must be trained to distinguish worms from the background. This is achieved by manually outlining worms on a test image and then running the Advanced Weka Segmentation plugin with the following filters selected; Gaussian blur, mean, Lipschitz, difference of Gaussians, variance and structure. The following parameters also have to be set: a sigma range of 2 to 16 pixels, membrane patch and thickness of 1 and 19 pixels. After training, the segmentation plugin is applied to the raw image file. Bona fide nematodes are distinguished from segmentation noise through particle analysis. The image stabilizer plugin is used to ensure that sequential scanned images are properly aligned. Once this pre-processing is completed, one of the followed procedures is carried out depending on the phenotype to be analyzed.

For behavior analysis a difference image is calculated that corresponds to the area of worm movement. The difference image is then converted to a binary (black & white) image through Hysteresis thresholding. The regions occupied by each worm in the initial image are overlaid with the corresponding area of the difference image. The fraction of overlapping pixels within each worm region that are white corresponds to the movement of that nematode. A worm is scored as dead if movement is less than 10%. To calculate worm length, individual worms have their curved morphological skeleton calculated through AnalyzeSkeleton. The length of this skeleton corresponds to the length of a straightened worm.

An abbreviated algorithm can be used to count worms as entities on the plate that move between sequential scans. This is achieved by aligning sequentially scanned images and generating a difference image. The contrast of the resulting difference image is enhanced by hysteresis, after which particle analysis is performed. This adaptation of WormScan is applicable to experiments that require routine counting of live worms such as fecundity and longevity experiments.

## Results

We demonstrate the utility of WormScan by quantifying the toxicological effects of exposure of *C. elegans* to phosphine gas. Additionally, we use our method to determine fecundity and lifespan in the absence of phosphine exposure as well as habituation to the light stimulus provided by the scanner. WormScan is also adaptable to other organisms of similar dimensions (Figure S1, S2).

**Figure 1:**
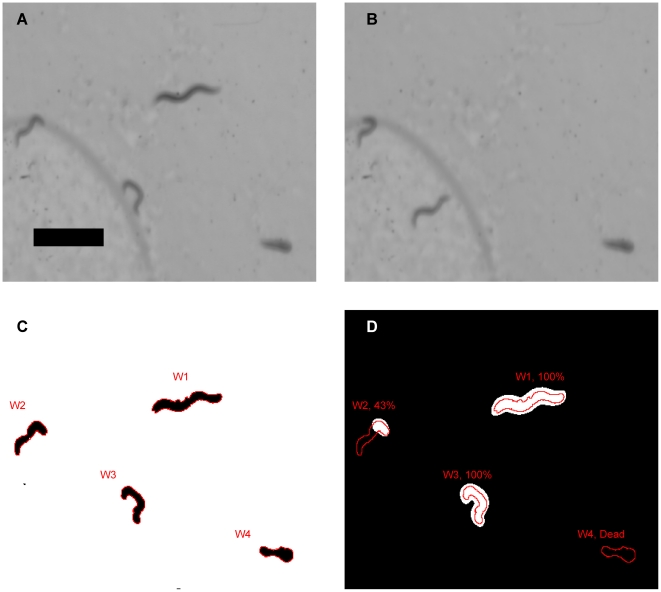
Processing and analysis of scanned images. (a) A 16-bit grey scale scan of a 5.5 cm petri dish containing N2 on NGM with OP50. Exposed to 200 ppm of phosphine for 24 hours with 48 hours of recovery. Only 4.5 by 4 mm crop is shown of the 5.5 cm. The black bar represents 1 mm, or 2400 dpi. (b) The sequential scan is taken 90 seconds after first. (c) Image segmentation with particle analysis, this distinguishes the nematodes from the background. Which are outlined with red lines, labeled W1–4. (d) Image difference of consecutive scans that has been thresholded with hysteresis. Where the worm positions are overlaid. This allows for calculation of both worm movement. Mortality is defined as less than less than 10% movement. Worm positions shown in red lines and labels commenting on level of movement.

Sequential scanned images are of sufficient resolution to visualize *C. elegans* that are greater than half a millimeter in length (Figure 1a,b). An adaptive local threshold is used to convert images to binary to allow clear segmentation of animals from the background. In these segmented images, *C. elegans* position is resolved through particle analysis (Figure 1c). The delineated positions of *C. elegans* are then evaluated against the calculated difference of the two scans. Where movement is quantified as the percent displacement of each nematode. A minimum displacement of 10% between scans is used as a threshold for mortality to ensure reproducibility (Figure 1c).

**Figure 2:**
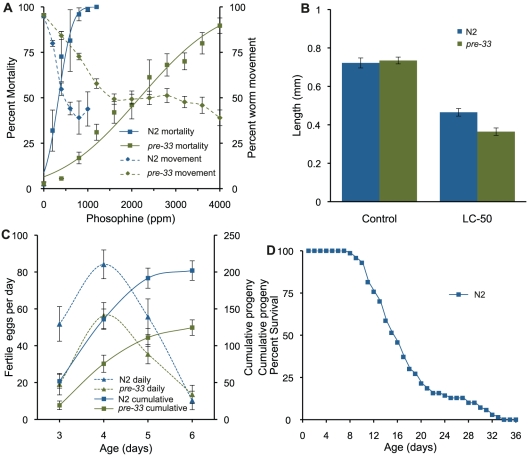
Toxicological end points of *C. elegans*. (a) Movement reduction and induced mortality resulting from phosphine exposure. Square boxes indicate mean mortality for a given concentration. Error bars denote the standard error of means from three biological replicates. The corresponding solid line is a probit regression of mortality, calculated in Mathematica 8.0. Dashed lines with diamond boxes represent the mean movement of the replicated experiments, where the error bars represent 95% confidence interval. Overall the LC_50_ of phosphine towards N2 is 337 and *pre-33* was found to be 2180 ppm. (b) Observed length differences between N2 and *pre-33* nematode strains after 48 hours recovery from phosphine exposure. Exposure was undertaken on L4 stage nematodes for 24 hours to phosphine at respective LC_50_ phosphine concentrations or air control. Error bars represent the standard error of mean from 3 biological replicates. (c) Fecundity of N2 and *pre-33* strains cultured in the absence of phosphine; the cumulative (**^___^**) or per day (—). Data was generated from three biological replicates of 6 worms each. The cumulative progeny on day six with 95% confidence is 202±13.2 for N2 and 125±10.5 for *pre-33*. (d) Lifespan of N2 cultured in the absence of phosphine. Mean lifespan was 17.3±0.6 from 3 trials of 48 nematodes per trial. Error is reported as the standard error of the means.

We used the flat bed scanner to compare mortality and behavior in response to phosphine exposure (Figure 2a). The mortality results are comparable to published data [36] and correspond closely with the observed behavioral inhibition.

Toxicity is sometimes reflected in altered growth parameters, which our algorithm is able to determine accurately. This is clearly seen in response to phosphine exposure, which results in growth inhibition of up to 50% in the *pre-33* mutant strain. It is interesting to note that resistance to phosphine induced mortality in *pre-33* is not reflected in a corresponding resistance to growth inhibition (Figure 2b).

We used the abbreviated WormScan algorithm in order to quantify the differences in fecundity between wild-type and the phosphine resistant mutant (Figure 2c). We observed the age of peak egg laying to be the same between the two strains within the resolution of the experiment. In contrast the cumulative number of eggs produced by N2 was nearly twice that of the mutant strain. The abbreviated algorithm was also used to monitor lifespan of wild-type *C. elegans*. The longevity of the N2 strain was found to be equivalent to published data (Figure 2d) [40].

**Figure 3:**
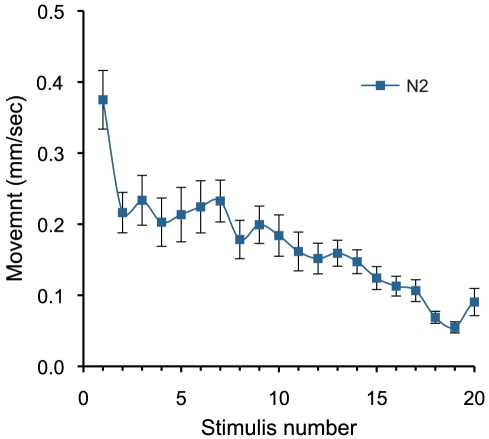
*C. elegans* phototaxis habituation. A classic habitation pattern is observed with continuous scanning with a 90-second interval for 20 intervals. Temperature on the scanner surfaces was monitored and found to be 20°C. The light response was measured in triplicate with 30 worms/plate.

One potential worry regarding the technique is that the scanning procedure itself could influence the outcome of the assays. To test this possibility, we subjected worms to continuous scanning. This produced a classic habitation pattern to light stimulus similar to that of tap habitation (Figure 3) [41] with significant habituation occurring after the first scan. Additionally, Continuous scanning over a period of 18 hours, did not induce mortality.

## Discussion

WormScan is a readily available, high-throughput image system based on a flatbed scanner. Allowing for the quantification of the four main toxicological endpoints of *C. elegans*. The main advantages of this high-throughput automated method are ease of setup and low cost. This protocol will help reduce user bias associated with manual counting [42].

The flatbed scanner produces sufficient light intensity to induce negative phototaxis in *C. elegans*. This produced a classic habituation pattern similar to that of physical stimulus [41]. It has also been shown that exposure from intense blue violet light can induce mortality in *C. elegans* [32]. While the scanner uses broad spectrum white light rather than blue violet, it is of an intensity that warrants investigation of its effect on the worms. We found that continuous scanning over 18 hours did not cause mortality. Therefore, other than habituation effects, the light of the scanner is unlikely to interfere with the accuracy of extended assays that require repeated scanning.

Behavior in higher organisms is very complex and thus difficult to quantify. In contrast, *C. elegans* exhibit a simpler behavioral repertoire that consisting primarily of changes in rate or direction of movement [31]. WormScan is restricted to changes in rate of movement, which is suitable for automation of assays that rely on the ability to respond to a physical stimulus such as tapping [17] or a chemical attractant [6]. Behavior as an indicator of toxicity can be 25 to 100 times more sensitive than mortality [43]. In this regard, behavior was shown to be a sensitive assay of phosphine toxicity.

Previous methods for quantifying mortality have much lower throughput than WormScan. Our method provides a uniform stimulus that induces a robust behavioral response great enough to allow accurate determination of mortality with high-throughput. Defining mortality as a threshold of less that 10% movement helps to eliminate false positives due to image noise. Using these parameters, mortality determination by WormScan is equivalent to that previously reported for phosphine exposure [35], [36], [37]. Using WormScan for lifespan determination eliminates the need for daily physical stimulation, which greatly reduces the potential for contamination.

WormScan has also been used to automate analysis of length and fecundity in *C. elegans*. Length of *C. elegans* can be determined by digitally straightening [44], [45]. This algorithm has been adopted by WormScan to expand the range of phenotypes that can be assessed. We used this to accurately determine the inhibition of growth of *C. elegans* due to phosphine exposure.

The other favorable properties of a scanner over microscopes with CCD cameras is greater optical density [42] and superior depth of field [46]. This allows for translucent *C. elegans* to be resolved from the background media. However, the resolution is not sufficient to resolve L1 stage nematodes.

*C. elegans* move at a rate of 0.5 mm/s [47], potentially resulting in significant movement between the required 90 seconds for a sequential scan. Current WormScan software can only measure movement up to one body length. This is not generally a problem as exposure to toxins and disease models often display phenotype of greatly decreased movement. Finer characteristics of nematode movement are not possible [48]. Which can be determined through low-throughput video microscope methods [9]. WormScan and these low-throughput camera based methods are complementary.

Image analysis uses the open source software, ImageJ. This allows improvements to be implemented to overcome current limitations. For example, the current implementation of WormScan requires non-overlapping nematodes for effective image analysis, which is achieved by limiting worm density to less than 150 individuals on a 5.5 cm diameter plate. However, worms can be digitally untangled, which could allow a higher density of worms per plate to be analyzed in future studies [49]. Furthermore, ImageJ was adapted to allow WormScan to observe similar sized species.

*C. elegans* is a model organism that has been applied to a wide range of studies. However, manual phenotypic analysis is labor intensive and time consuming. WormScan automates high-throughput scoring of the most widely used whole-organism assays performed on *C. elegans*. This affordable, open platform will enable wide adoption with significant potential to reduce data variability between labs [9].

## Supporting Information

Figure S1 **Size quantification of** ***Tribolium castaneum*** **.**
(PDF)

Figure S2 **Quantification of** ***Trichogramma*** **.**
(PDF)

Tutorial S1 This tutorial package describes the minimal system requirements as well as how to access and set up the required open source software on your computer (in the Tutorial.docx file). The Tutorial.docx file also contains a step-by-step description of how to conduct the analyses described in the paper. To run the tutorial, you will use the included custom scripts (the 7 .ijm files) specific to the WormScan image analysis as well as the included set of demonstration images (the 5 .tif files). To begin the tutorial, open the Tutorial.docx file and follow the instructions. To Assist with trouble shooting, a folder labeled Sample_results is included as an example of the results you should expect to obtain from the analysis when you use the provided training file, RoiSet.zip.
(ZIP)
