# Supplementary material for: WormScan: A Technique for High-Throughput Phenotypic Analysis of Caenorhabditis elegans
Source: PLoS One. 2012 Mar 23;7(3):e33483. doi: 10.1371/journal.pone.0033483 (PMC3311640; doi:10.1371/journal.pone.0033483)
Supplement: Figure S1 — Size quantification of Tribolium castaneum . (PDF) [file pone.0033483.s001.pdf]

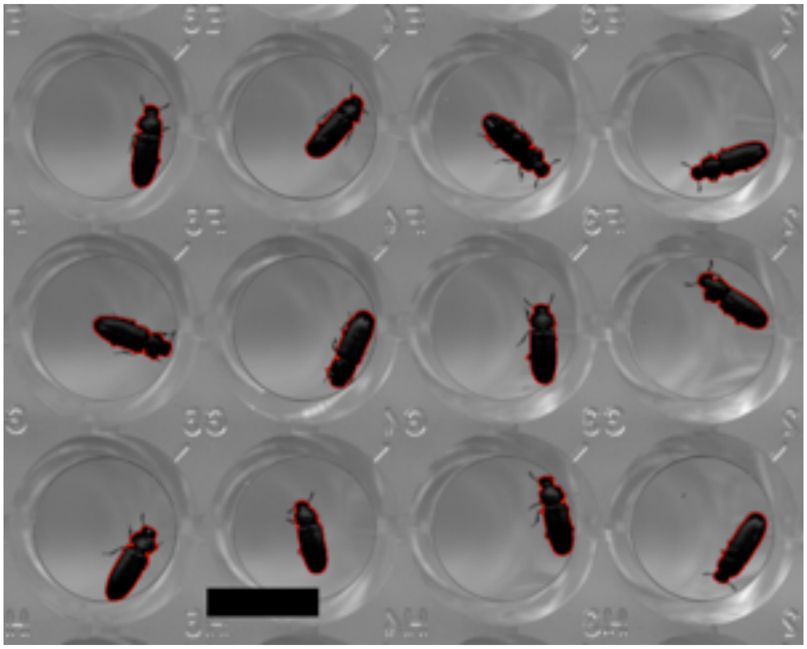

Figure S1. Size quantification of *Tribolium castaneum.* Individual live beetles were placed in a clearly flat-bottom 96-well microtitre plates. Beetles were anaesthetized with CO_2 for 5 minutes in a sealed desiccation chamber and the plates were scanned using reflective mode, 600 dpi and 8-bit grey-scale. The black bar represents 5 mm. Adaptive local threshold to differentiate beetles from the background was used. Particle analysis was used to extract beetles from the binary image. Limbs were removed using a binary erode and expand script retaining an outline of the beetle head thorax and abdomen (script not published). Measurements were taken on the resulting processed image to determine area, width and length.
