# Supplementary material for: WormScan: A Technique for High-Throughput Phenotypic Analysis of Caenorhabditis elegans
Source: PLoS One. 2012 Mar 23;7(3):e33483. doi: 10.1371/journal.pone.0033483 (PMC3311640; doi:10.1371/journal.pone.0033483)
Supplement: Figure S2 — Quantification of Trichogramma . (PDF) [file pone.0033483.s002.pdf]

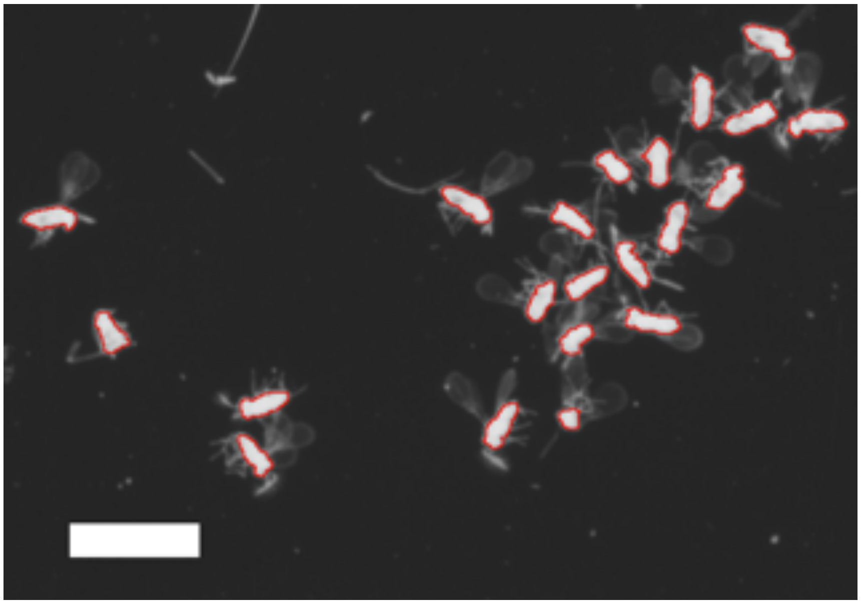

Figure S2. Quantification of *Trichogramma.* Approximately 1,000 Stingless wasps were placed in a 8 cm petri dish containing 70% ethanol and were scanned using transmission mode, 2400 dpi at 8-bit grey scale. The white bar represents 1 mm. Adaptive local threshold to differentiate the wasps from the background was used. Particle analysis was used to extract wasps from the binary image.
