# Supplementary material for: WormScan: A Technique for High-Throughput Phenotypic Analysis of Caenorhabditis elegans
Source: PLoS One. 2012 Mar 23;7(3):e33483. doi: 10.1371/journal.pone.0033483 (PMC3311640; doi:10.1371/journal.pone.0033483)
Supplement: Tutorial S1 — This tutorial package describes the minimal system requirements as well as how to access and set up the required open source software on your computer (in the Tutorial.docx file). The Tutorial.docx file also contains a step-by-step description of how to conduct the analyses described in the paper. To run the tutorial, you will use the included custom scripts (the 7 .ijm files) specific to the WormScan image analysis as well as the included set of demonstration images (the 5 .tif files). To begin the tutorial, open the Tutorial.docx file and follow the instructions. To Assist with trouble shooting, a folder labeled Sample_results is included as an example of the results you should expect to obtain from the analysis when you use the provided training file, RoiSet.zip. (ZIP) [file pone.0033483.s003.zip › tutorial/tutorial.pdf]

WormScan Tutorial

WormScan: A Technique for High-Throughput Phenotypic Analysis of *Caenorhabditis elegans* PloS ONE, 2012. Briefly, the outline of the image processing procedure is as follows:

1) Hardware Requirements:

The recommended system requirements are 16 GB of memory and i7 quad-core Intel Core i7 processor for segmentation analysis. A high-end consumer grade scanner is required for image acquisition. We recommend an Epson V700 flatbed transmission film scanner. The software scripts in this archive contain the core functionality. With modification, these scripts can be used to analyse large image sets. We have provided a tutorial image set for demonstration purposes.

2) Installation of software:

The worm movement algorithm requires installation of;

- Fiji (with ImageJ 1.46a or newer) image analysis software
  http://fiji.sc/wiki/index.php/Downloads,
    - Run the updates to get the latest version of Advanced Weka Segmentation
        - Fiji -> help -> update
    - Restart FIJI
- Image stabilizer plug-in
  http://www.kangli.org/code/Image_Stabilizer.html
  (latest release - 2009/06/12)
  Download Image_Stabilizer.java and place into fiji.app/plugins folder
- Hysteresis thresholding (included in Image Edge plug-in)
  http://imagejdocu.tudor.lu/doku.php?id=plugin:filter:edge_detection:start
  Download image_edge.jar and place into fiji.app/plugins folder
  [To find the Plugins folder on a Mac - right click on the Fiji application icon and select "show package contents"]

3) Overview of WormScan outputs

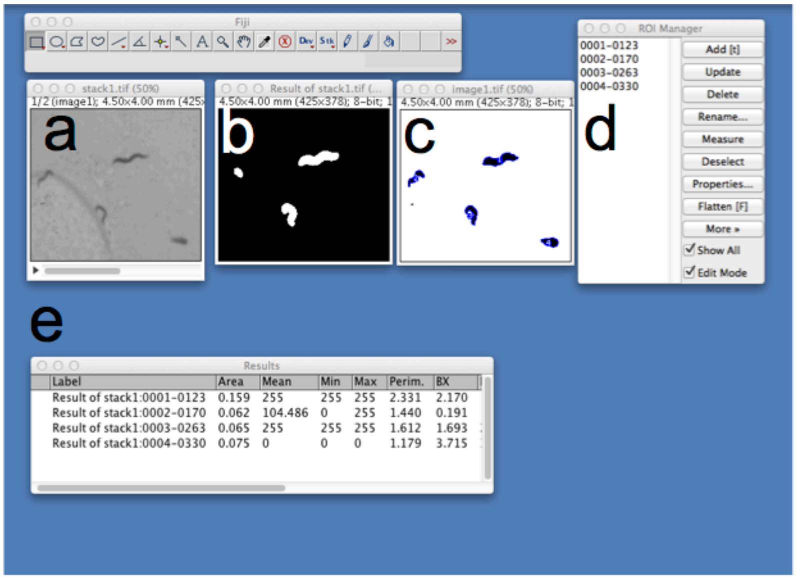

Figure 1: Mortality and movement analysis from sequentially scanned images.

a) A two-image stack of sequential scans.

b) Image difference between the two stacked images.

c) Image segmentation output, with nematodes distinguished from the background.
d) Region Of Interest (ROI) window showing labels corresponding to each identified worm.
e) Data table showing the label from (d) for each worm identified in (c) with movement calculated from the difference image (b).
These files can be found in the Mortality and Movement Results folder within the Sample_Results folder. The file names are as follows: a, stack1.tif; b, Result of stack1.tif; c, image1.tif; d, RoiSet.zip; e, Results.xls. The important output file is Result.xls, which contains the data output of the analysis. Two additional files that are generated but are not shown in Figure 1 are slice1.tif and classifier.model. These two files are required for intermediate steps in the analysis.

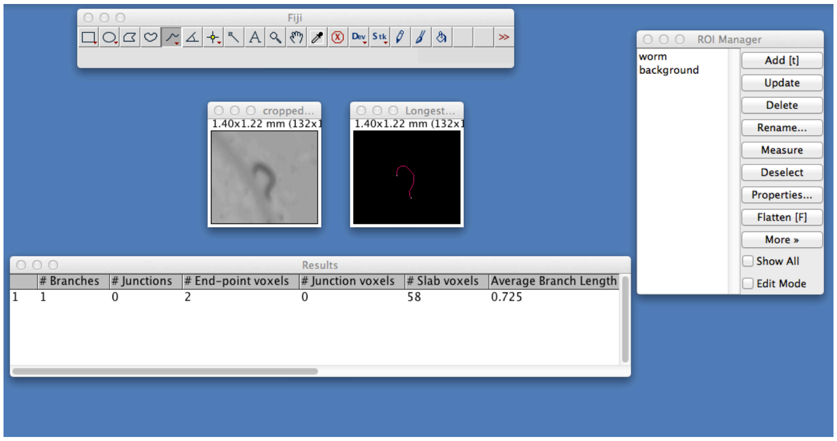

Figure 2: Length determination of a single worm.
The image on the left shows the original worm. The image on the right shows the morphological skeleton of the worm. The ROI table is on the far right. The length is shown in the results table at the bottom of the figure. These files are in the Length Results folder within Sample_Results folder.

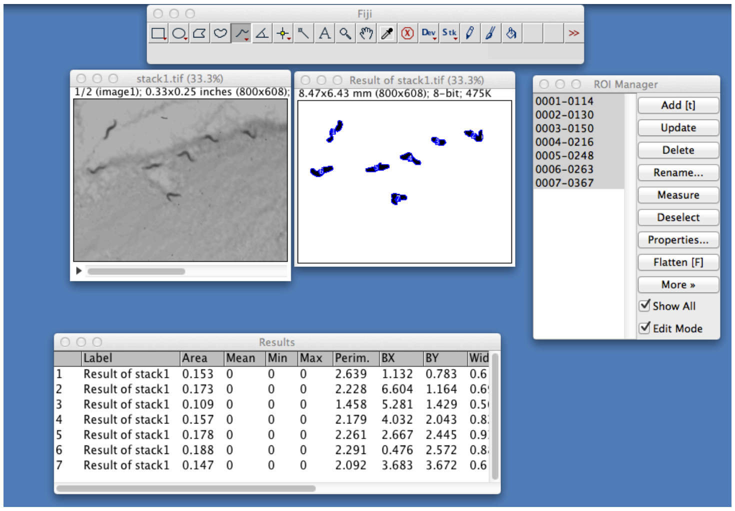

Figure 3: The abbreviated version of WormScan for counting live worms. Two sequential scans are used to generate the image stack on the left. The second image shows living worms based on movement between the two sequential scans that make up the image stack. Labels corresponding to mobile worm are shown in the ROI. Live worms are also listed in the results table together with their sizes in mm^2. These files are in the Abbreviated Version folder within Sample_Results folder.

4) Tutorials

[Importantly, for this tutorial the following steps have to be carried out in the precise order as shown]

4.1) Pre-analysis preparation - training the automatic worm recognition (image segmentation) tool:

- Open Fiji with installed plug-ins
- Open cropped_image.tif in tutorial folder.
- Open RioSet.zip in tutorial folder.
    - [This is an ROI file corresponding to cropped_image.tif. We generated this file for you for training purposes by manually selecting the worm. Normally, you would have to do this selection step yourself.]
- To start the Advanced Weka Segmentation, go to:
    - plug-ins > segmentation > Advanced Weka Segmentation
    - In the ROI Manager, highlight "worm" and click "Add to class 1" in the Weka segmentation window
    - In the ROI Manager, highlight "background" and click "Add to class 2"
- To apply the settings for Advanced Weka Segmentation
    - Click the settings button on the left and select the following options; Gaussian blur, mean, Lipschitz, difference of Gaussians, variance and structure.
    - set the sigma range to 2 to 16 pixels
    - set the membrane patch to a thickness of 1 and 19 pixels,
    - set the algorithm, fastRandomForest –l 200 –K 2 -S
- Click Okay on settings window
- Click "Train classifier" button
    - The layout of FIJI should look like (Figure 4)
- Click save classifier model into the tutorial folder, as classifier.model in the tutorial folder.

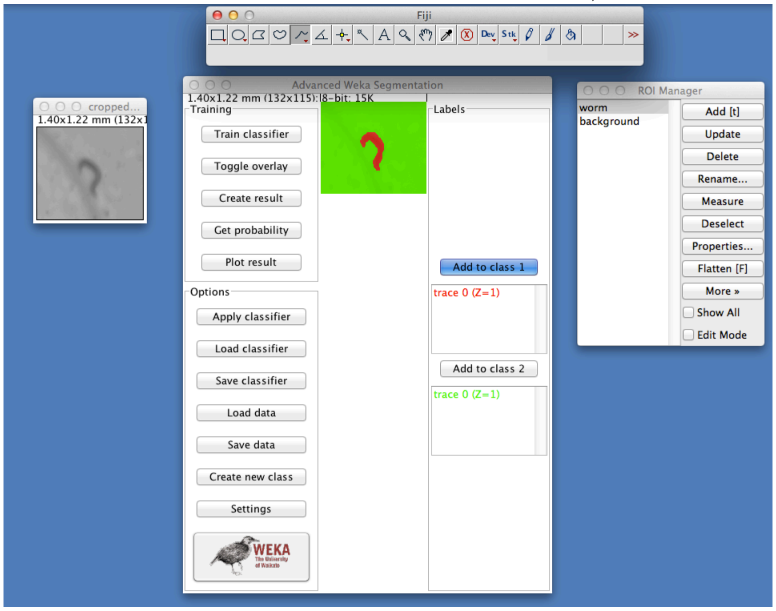

Figure 4: Training the image segmentation plug-in. Prior to image segmentation, the software has to be trained to distinguish worms from background image noise. To achieve this, one or more worms are manually selected to provide the software with an example of what it is to recognize. This produces a classifier that can be applied to images for analysis. Shown in the figure is the original image that contains a single worm, as well as the manually segmented image (colour coded red for the worm and green for the background) already loaded into the Advanced Weka Segmentation plug-in. The ROI manager is shown on the right.

- Close Advanced Weka Segmentation window
- Close cropped_image.tif and close the ROI manager

4.2) Align sequential scans and calculate image difference:

- Open scan1.tif
- Open scan2.tif
- Run macro M1.ijm
    - Plugins > Macro > Run.. . highlight macro M1.ijm, click "open"
- Save slice1.tif in the tutorial folder for later use

4.3) Apply the automatic worm recognition (image segmentation):

- Open macro M2.ijm
- Run macro M2.ijm
    - Plugins > Macro > Run.. . highlight macro M2.ijm, click "open"
- Click "Load Classifier" in Advanced Weka Segmentation window - classifier.model
    - Generated earlier in step 4.1 and saved to the tutorial folder
- Click "Apply classifier", select slice1.tif
    - That derived in step 4.2 and saved to the tutorial folder
- Click "No" to the apply probably maps

4.4) Quantify movement and determine mortality:

- Run macro M3.ijm
    - Plugins > Macro > Run.. . highlight macro M3.ijm, click "open"
- Run macro M4.ijm
    - Plugins > Macro > Run.. . highlight macro M4.ijm, click "open"
- Save results table
- Open results table with spreadsheet software.
    - Take the "mean" column and divide it by 255
        - This value represents the percentage moved of an individual worm.
        - If this value is less than 0.1 (or 10%) the worm is classified as dead
        - Also if this value is greater than 0.1 (or 10%) this is percent movement of alive worm

5) Measure length of a curved worm:

- Close all open windows in FIJI.
- Open image cropped_image.tif
- Open roiset.zip
- Run macro_measure.ijm
    - Plugins > Macro > Run.. . highlight macro_measure.ijm, click "open"
- The worm length is the "maximum branch length" in the results window.
    - The measurement units given for the worm are mm

6) Abbreviated WormScan method for counting live worms that move between scans

- Close all existing windows in FIJI.
- Open movement_scan1.tif
- Open movement_scan2.tif
- Run macro_abbreviated.ijm
    - Plugins > Macro > Run.. . highlight macro_abbreviated.ijm, click "open"
- Run macro abbreviated
- The results table will display details about each individual live worm, such as area in mm^2
