# Supplementary figures and images for: WormScan: A Technique for High-Throughput Phenotypic Analysis of Caenorhabditis elegans
Source: PLoS One. 2012 Mar 23;7(3):e33483. doi: 10.1371/journal.pone.0033483 (PMC3311640; doi:10.1371/journal.pone.0033483)

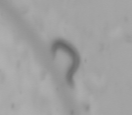

Supplement: Tutorial S1 — This tutorial package describes the minimal system requirements as well as how to access and set up the required open source software on your computer (in the Tutorial.docx file). The Tutorial.docx file also contains a step-by-step description of how to conduct the analyses described in the paper. To run the tutorial, you will use the included custom scripts (the 7 .ijm files) specific to the WormScan image analysis as well as the included set of demonstration images (the 5 .tif files). To begin the tutorial, open the Tutorial.docx file and follow the instructions. To Assist with trouble shooting, a folder labeled Sample_results is included as an example of the results you should expect to obtain from the analysis when you use the provided training file, RoiSet.zip. (ZIP) [file pone.0033483.s003.zip › tutorial/cropped_image.tif]

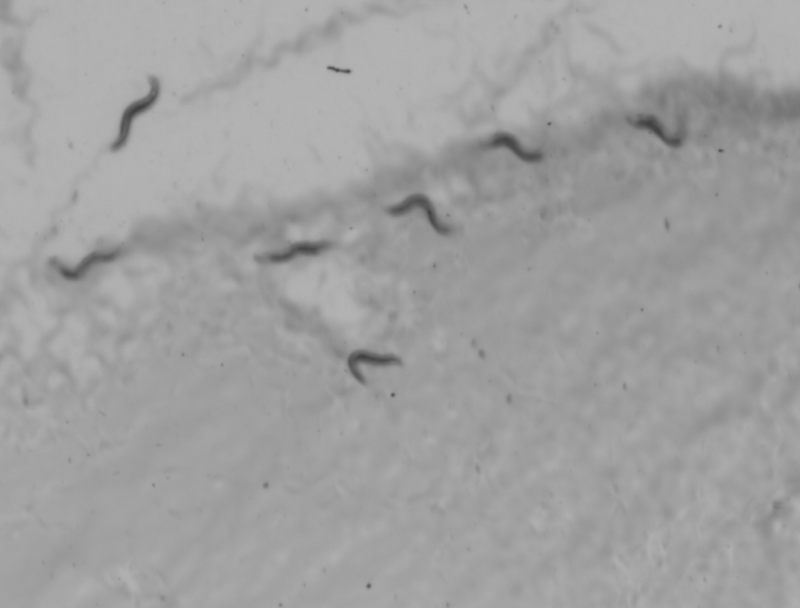

Supplement: Tutorial S1 — This tutorial package describes the minimal system requirements as well as how to access and set up the required open source software on your computer (in the Tutorial.docx file). The Tutorial.docx file also contains a step-by-step description of how to conduct the analyses described in the paper. To run the tutorial, you will use the included custom scripts (the 7 .ijm files) specific to the WormScan image analysis as well as the included set of demonstration images (the 5 .tif files). To begin the tutorial, open the Tutorial.docx file and follow the instructions. To Assist with trouble shooting, a folder labeled Sample_results is included as an example of the results you should expect to obtain from the analysis when you use the provided training file, RoiSet.zip. (ZIP) [file pone.0033483.s003.zip › tutorial/movement_scan1.tif]

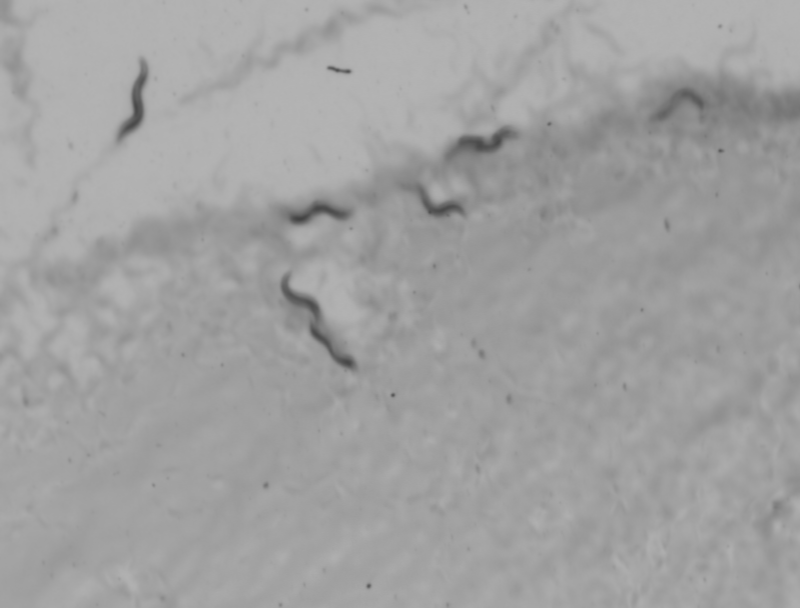

Supplement: Tutorial S1 — This tutorial package describes the minimal system requirements as well as how to access and set up the required open source software on your computer (in the Tutorial.docx file). The Tutorial.docx file also contains a step-by-step description of how to conduct the analyses described in the paper. To run the tutorial, you will use the included custom scripts (the 7 .ijm files) specific to the WormScan image analysis as well as the included set of demonstration images (the 5 .tif files). To begin the tutorial, open the Tutorial.docx file and follow the instructions. To Assist with trouble shooting, a folder labeled Sample_results is included as an example of the results you should expect to obtain from the analysis when you use the provided training file, RoiSet.zip. (ZIP) [file pone.0033483.s003.zip › tutorial/movement_scan2.tif]

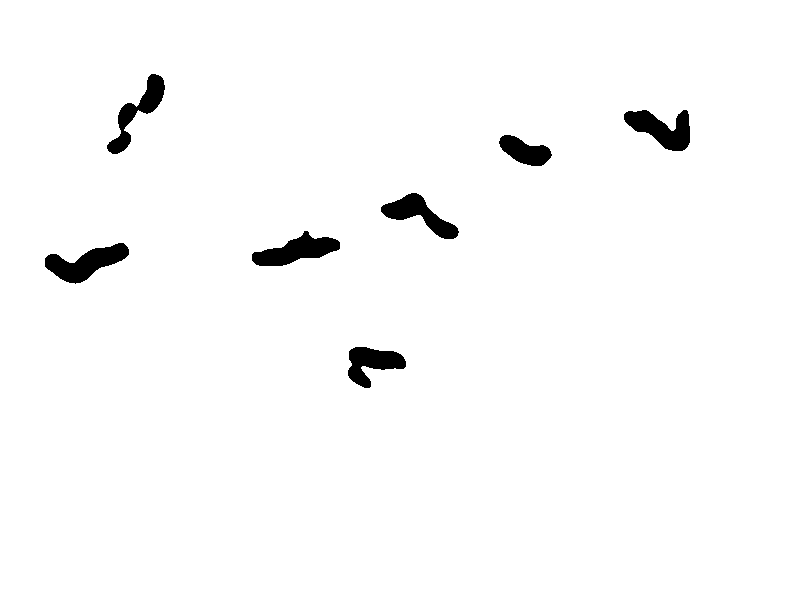

Supplement: Tutorial S1 — This tutorial package describes the minimal system requirements as well as how to access and set up the required open source software on your computer (in the Tutorial.docx file). The Tutorial.docx file also contains a step-by-step description of how to conduct the analyses described in the paper. To run the tutorial, you will use the included custom scripts (the 7 .ijm files) specific to the WormScan image analysis as well as the included set of demonstration images (the 5 .tif files). To begin the tutorial, open the Tutorial.docx file and follow the instructions. To Assist with trouble shooting, a folder labeled Sample_results is included as an example of the results you should expect to obtain from the analysis when you use the provided training file, RoiSet.zip. (ZIP) [file pone.0033483.s003.zip › tutorial/sample_results/Abbreviated Version/Result of stack1.tif]

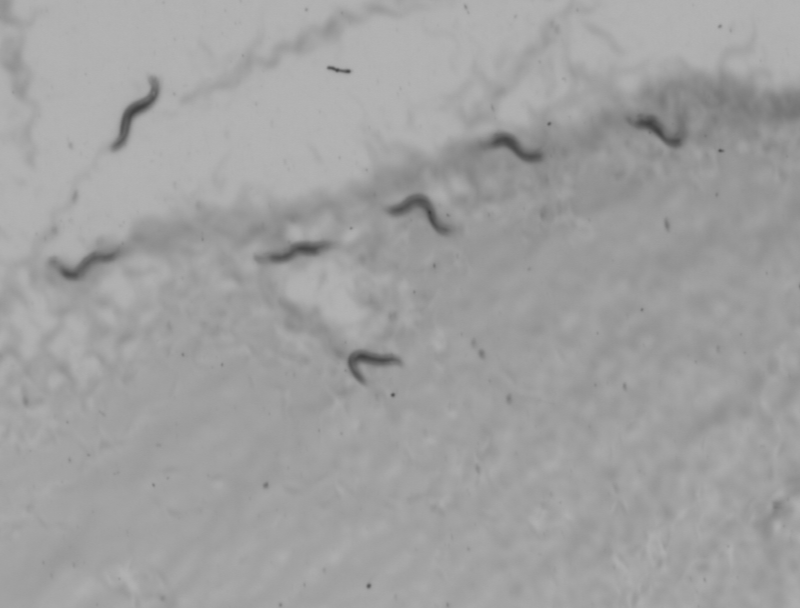

Supplement: Tutorial S1 — This tutorial package describes the minimal system requirements as well as how to access and set up the required open source software on your computer (in the Tutorial.docx file). The Tutorial.docx file also contains a step-by-step description of how to conduct the analyses described in the paper. To run the tutorial, you will use the included custom scripts (the 7 .ijm files) specific to the WormScan image analysis as well as the included set of demonstration images (the 5 .tif files). To begin the tutorial, open the Tutorial.docx file and follow the instructions. To Assist with trouble shooting, a folder labeled Sample_results is included as an example of the results you should expect to obtain from the analysis when you use the provided training file, RoiSet.zip. (ZIP) [file pone.0033483.s003.zip › tutorial/sample_results/Abbreviated Version/stack1.tif]

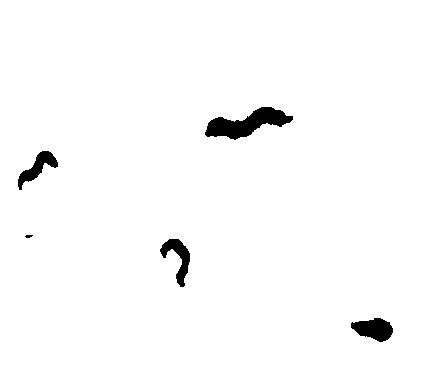

Supplement: Tutorial S1 — This tutorial package describes the minimal system requirements as well as how to access and set up the required open source software on your computer (in the Tutorial.docx file). The Tutorial.docx file also contains a step-by-step description of how to conduct the analyses described in the paper. To run the tutorial, you will use the included custom scripts (the 7 .ijm files) specific to the WormScan image analysis as well as the included set of demonstration images (the 5 .tif files). To begin the tutorial, open the Tutorial.docx file and follow the instructions. To Assist with trouble shooting, a folder labeled Sample_results is included as an example of the results you should expect to obtain from the analysis when you use the provided training file, RoiSet.zip. (ZIP) [file pone.0033483.s003.zip › tutorial/sample_results/Mortality and Movement Results/image1.tif]

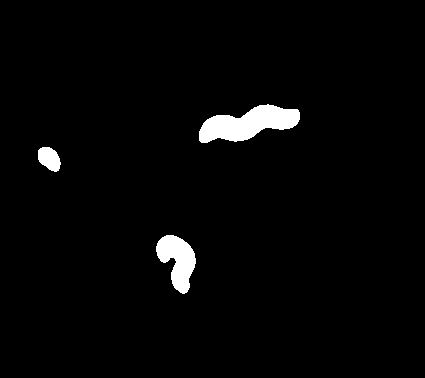

Supplement: Tutorial S1 — This tutorial package describes the minimal system requirements as well as how to access and set up the required open source software on your computer (in the Tutorial.docx file). The Tutorial.docx file also contains a step-by-step description of how to conduct the analyses described in the paper. To run the tutorial, you will use the included custom scripts (the 7 .ijm files) specific to the WormScan image analysis as well as the included set of demonstration images (the 5 .tif files). To begin the tutorial, open the Tutorial.docx file and follow the instructions. To Assist with trouble shooting, a folder labeled Sample_results is included as an example of the results you should expect to obtain from the analysis when you use the provided training file, RoiSet.zip. (ZIP) [file pone.0033483.s003.zip › tutorial/sample_results/Mortality and Movement Results/Result of stack1.tif]

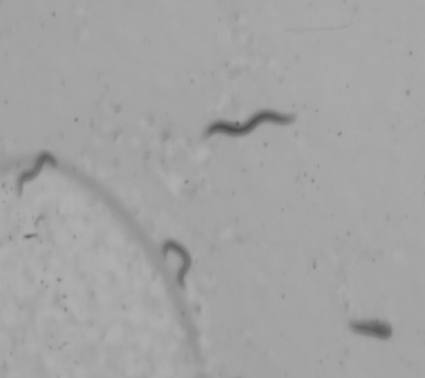

Supplement: Tutorial S1 — This tutorial package describes the minimal system requirements as well as how to access and set up the required open source software on your computer (in the Tutorial.docx file). The Tutorial.docx file also contains a step-by-step description of how to conduct the analyses described in the paper. To run the tutorial, you will use the included custom scripts (the 7 .ijm files) specific to the WormScan image analysis as well as the included set of demonstration images (the 5 .tif files). To begin the tutorial, open the Tutorial.docx file and follow the instructions. To Assist with trouble shooting, a folder labeled Sample_results is included as an example of the results you should expect to obtain from the analysis when you use the provided training file, RoiSet.zip. (ZIP) [file pone.0033483.s003.zip › tutorial/sample_results/Mortality and Movement Results/slice1.tif]

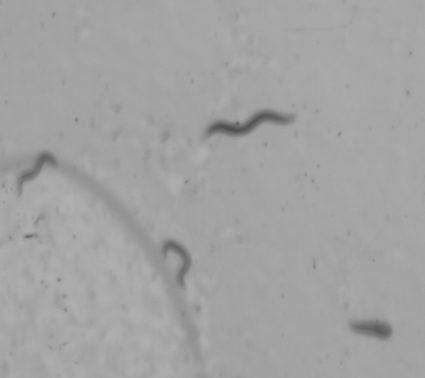

Supplement: Tutorial S1 — This tutorial package describes the minimal system requirements as well as how to access and set up the required open source software on your computer (in the Tutorial.docx file). The Tutorial.docx file also contains a step-by-step description of how to conduct the analyses described in the paper. To run the tutorial, you will use the included custom scripts (the 7 .ijm files) specific to the WormScan image analysis as well as the included set of demonstration images (the 5 .tif files). To begin the tutorial, open the Tutorial.docx file and follow the instructions. To Assist with trouble shooting, a folder labeled Sample_results is included as an example of the results you should expect to obtain from the analysis when you use the provided training file, RoiSet.zip. (ZIP) [file pone.0033483.s003.zip › tutorial/sample_results/Mortality and Movement Results/stack1.tif]

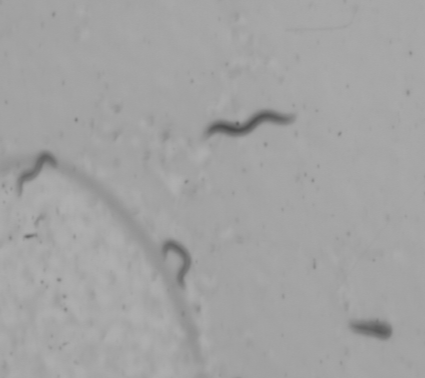

Supplement: Tutorial S1 — This tutorial package describes the minimal system requirements as well as how to access and set up the required open source software on your computer (in the Tutorial.docx file). The Tutorial.docx file also contains a step-by-step description of how to conduct the analyses described in the paper. To run the tutorial, you will use the included custom scripts (the 7 .ijm files) specific to the WormScan image analysis as well as the included set of demonstration images (the 5 .tif files). To begin the tutorial, open the Tutorial.docx file and follow the instructions. To Assist with trouble shooting, a folder labeled Sample_results is included as an example of the results you should expect to obtain from the analysis when you use the provided training file, RoiSet.zip. (ZIP) [file pone.0033483.s003.zip › tutorial/scan1.tif]

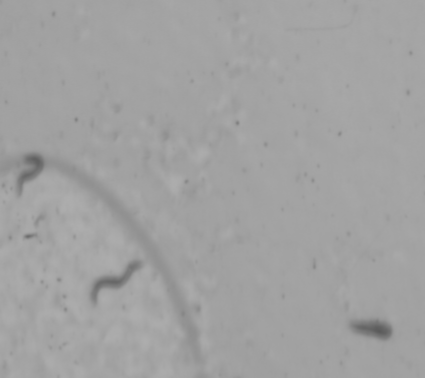

Supplement: Tutorial S1 — This tutorial package describes the minimal system requirements as well as how to access and set up the required open source software on your computer (in the Tutorial.docx file). The Tutorial.docx file also contains a step-by-step description of how to conduct the analyses described in the paper. To run the tutorial, you will use the included custom scripts (the 7 .ijm files) specific to the WormScan image analysis as well as the included set of demonstration images (the 5 .tif files). To begin the tutorial, open the Tutorial.docx file and follow the instructions. To Assist with trouble shooting, a folder labeled Sample_results is included as an example of the results you should expect to obtain from the analysis when you use the provided training file, RoiSet.zip. (ZIP) [file pone.0033483.s003.zip › tutorial/scan2.tif]
